# Effects of Supplementation with a Microalgae Extract from *Phaeodactylum tricornutum* Containing Fucoxanthin on Cognition and Markers of Health in Older Individuals with Perceptions of Cognitive Decline

**DOI:** 10.3390/nu16172999

**Published:** 2024-09-05

**Authors:** Choongsung Yoo, Jonathan Maury, Drew E. Gonzalez, Joungbo Ko, Dante Xing, Victoria Jenkins, Broderick Dickerson, Megan Leonard, Landry Estes, Sarah Johnson, Jisun Chun, Jacob Broeckel, Rémi Pradelles, Ryan Sowinski, Christopher J. Rasmussen, Richard B. Kreider

**Affiliations:** 1 Exercise & Sport Nutrition Lab., Department of Kinesiology and Sports Management, Texas A&M University, College Station, TX 77843, USA; choongsungyoo@tamu.edu (C.Y.); dg18@tamu.edu (D.E.G.); joungboko10@tamu.edu (J.K.); dantexing@tamu.edu (D.X.); victoria.jenkins@tamu.edu (V.J.); dickersobl5@email.tamu.edu (B.D.); meganleonard10@tamu.edu (M.L.); landry.estes@tamu.edu (L.E.); sjohnson2216@tamu.edu (S.J.); chunjs3112@tamu.edu (J.C.); broeckelj@tamu.edu (J.B.); rjs370@tamu.edu (R.S.); crasmussen@tamu.edu (C.J.R.); 2 Research & Development Department, Microphyt, 34670 Baillargues, France; jonathan.maury@microphyt.eu (J.M.); remi.pradelles@microphyt.eu (R.P.)

**Keywords:** fucoxanthin, memory, reaction time, attention, vigilance, quality of life

## Abstract

*Phaeodactylum tricornutum* (*PT*) is a microalgae extract that contains fucoxanthin and has been shown to enhance cognitive function in younger populations. The present study assessed if *PT* supplementation affects cognition in healthy, young-old, physically active adults with self-perceptions of cognitive and memory decline. Methods: Forty-three males and females (64.3 ± 6.0 years, 79.8 ± 16.0 kg, 27.0 ± 4.0 kg/m^2^) with perceptions of cognitive and memory decline completed the double-blind, randomized, parallel-arm, placebo-controlled intervention clinical trial. Participants were counterbalanced by sex and BMI and randomly allocated to their respective 12-week supplementation interventions, which were either the placebo (PL) or 1100 mg/day of *PT* containing 8.8 mg of fucoxanthin (FX). Fasting blood samples were collected, and cognitive assessments were performed during the testing session at 0, 4, and 12 weeks of intervention. The data were analyzed by multivariate and univariate general linear model (GLM) analyses with repeated measures, pairwise comparisons, and mean changes from baseline analysis with 95% confidence intervals (CIs) to assess the clinical significance of the findings. Results: FX supplementation significantly affected (*p* < 0.05) or exhibited tendencies toward significance (*p* > 0.05 to *p* < 0.10 with effect sizes ranging from medium to large) for word recall, picture recognition reaction time, Stroop color–word test, choice reaction time, and digit vigilance test variables. Additionally, FX supplementation promoted a more consistent clinical improvement from baseline values when examining mean changes with 95% CIs, although most differences were seen over time rather than between groups. Conclusions: The results demonstrate some evidence that FX supplementation can improve working and secondary memory, vigilance, attention, accuracy, and executive function. There was also evidence that FX promoted more positive effects on insulin sensitivity and perceptions about sleep quality with no negative effects on clinical blood panels or perceived side effects. Additional research should investigate how FX may affect cognition in individuals perceiving memory and cognitive decline. Registered clinical trial #NCT05759910.

## 1. Introduction

Cognitive processes refer to a series of brain functions that facilitate acquiring, processing, storing, and utilizing information obtained from the environment [1]. Age-associated cognitive decline refers to a non-pathological reduction in cognition, including speed, attention, information processing, and short-term/working memory [2]. This includes cognitive and executive function domains, such as sensation and perception, attention, psychomotor function, processing speed, memory, logic and reasoning, problem-solving, and language and verbal ability [3,4]. Cognitive and executive function changes naturally occur as we age because of changes in brain anatomy, physiology, and health and lifestyle-related risk factors [5]. However, the development of mild cognitive impairment (MCI) and dementia are not inherent aspects of aging. MCI has generally been considered as the stage before a diagnosis of dementia [4], while age-associated cognitive decline is commonly associated with neurodegenerative diseases (e.g., Alzheimer’s disease) and vascular cognitive impairment [6,7]. 

Various pharmacological, nutritional, and behavioral interventions have been examined to sustain or improve cognition as people age [7,8]. These interventions include increasing physical activity [8,9], social interaction [10], and participating in intellectually stimulating activities [11]. Dietary interventions such as adherence to the Mediterranean diet [12,13,14] and supplementation of omega-3 fatty acids [15,16,17,18,19], folic acid [20,21], vitamin D [22], a yogurt-like drink containing omega-3 fatty acids, choline, phospholipids, folic acid, antioxidants [23,24,25], and others [7,16,23,26,27,28,29,30] have been studied in an attempt to help maintain cognitive function in individuals experiencing memory issues or diagnosed with MCI and/or dementia. While the interaction between nutrition and an aging brain is not fully understood [31], reductions in cerebral blood flow related to atherosclerosis and arterial plaque formation [32], mitochondrial dysfunction resulting in part from oxidative stress [33], and inflammation [34] can negatively affect aging and cognition [30,35,36]. Reducing oxidative stress and inflammation as we age has been suggested as a primary way to help maintain cognitive function and delay the onset of memory and cognitive decline [16].

Fucoxanthin, a carotenoid procured from marine brown seaweed and microalgae, can traverse the blood–brain barrier and exert antioxidant and anti-inflammatory benefits [37,38]. Basic research studies have shown that fucoxanthin feeding attenuated cognitive impairment in aging mice [39,40]. Additionally, reports have demonstrated that marine algae and fucoxanthinol can help manage weight gain [38,41], lower blood lipids [41,42,43], and control blood glucose primarily by mediating inflammatory pathways [41,44]. We completed two clinical trials examining the impact of fucoxanthin supplementation on health and cognitive performance. First, Leonard et al. [45] reported that acute and 30 days of *PT*-derived fucoxanthin supplementation (4.4 mg/d) with guarana (containing 40–55 mg/d of caffeine) improved impulsiveness, reasoning, reaction times, executive control, learning, and cognitive flexibility among young adult e-gamers. That study provided evidence that acute and short-term fucoxanthin can affect cognitive function. Second, Dickerson et al. [46] found that fucoxanthin ingestion (4.4 mg/d for 3 months) extracted from *Phaeodactylum Tricornutum* (*PT*) maintained bone mass, augmented bone density, and promoted exercise and diet program adherence in overweight pre-menopausal females, leading to more favorable changes in aerobic capacity, blood lipids, and perceptions about improved sleep, functional capacity, and quality of life. That study demonstrated that fucoxanthin supplementation may have exercise, health, sleep, and cognitive benefits that would theoretically benefit older individuals. 

The present study examined whether fucoxanthin supplementation (8.8 mg/d for 12 weeks) from *PT* extract affects cognition and/or health parameters in healthy, young-old individuals beginning to experience memory or cognitive decline. We hypothesized that fucoxanthin supplementation would improve this population’s cognitive function and memory indices. Additionally, fucoxanthin supplementation would be easily tolerated and not negatively affect health markers in this population. The following overviews the study details and results and discusses the findings’ implications.

## 2. Methods

### 2.1. Research Design

This study was executed as a double-blind, randomized, parallel-arm, placebo-controlled intervention clinical trial conducted in a university research setting. The independent variable was nutritional supplementation. The primary outcome was cognitive function measures. The secondary outcomes were changes in subjective ratings of perceived stress and mood symptoms, sleep quality questionnaire responses, blood markers of health (e.g., clinical chemistry panels, glucose homeostasis, inflammatory markers), and self-reported subjective side effects. All testing and analyses were performed at the Exercise and Sport Nutrition Laboratory (ESNL) and biochemistry facilities within the Human Clinical Research Faculty at Texas A&M University. 

### 2.2. Study Participants 

Healthy, free-living, young-old adults between 55 and 75 years with a body mass index (BMI) between 18.5 and 35 kg/m^2^ were recruited for this 12-week clinical trial. Recruitment mass emails, flyers, and website advertisements were used. Interested individuals were pre-screened for eligibility via an online questionnaire before attending a familiarization session, during which all study protocols and procedures were reviewed, written informed consent was provided, health and medical histories were obtained, and physical examinations were completed to determine eligibility. Eligible participants had to meet the following age-associated memory impairment (AAMI) criteria as outlined by the National Institute of Mental Health: (1) a score of 24 or more on the Mini-Mental State Examination (MMSE), suggesting the absence of dementia, and (2) a score of 25 or higher on the Memory Complaint Questionnaire (MAC-Q), indicative if perceptions of subjective memory complaints. Individuals were excluded if they met any of the following criteria: (1) used cognition-altering medications within the past two weeks; (2) had abnormal clinical laboratory tests that may affect the study outcome; (3) had cancer or a history of cancer (excluding nonmelanoma skin cancer); (4) had uncontrolled hypertension and/or diabetes; (5) had a history of depression (the past year) or used psychotropic medications within one month of the screening; (6) had a history of alcoholism or substance abuse within the last 12 months; (7) were a heavy smoker (>1 pack/day within the past three months); and (8) were knowingly allergic to the ingredients of the supplement product (Brainphyt™, Microphyt, Baillargues, France) or placebo (maltodextrin). The University Institutional Review Board (IRB2021-1360F) approved this study, which was conducted by the Declaration of Helsinki and was registered with ClinicalTrials.gov (NCT05759910).

**Figure 1:**
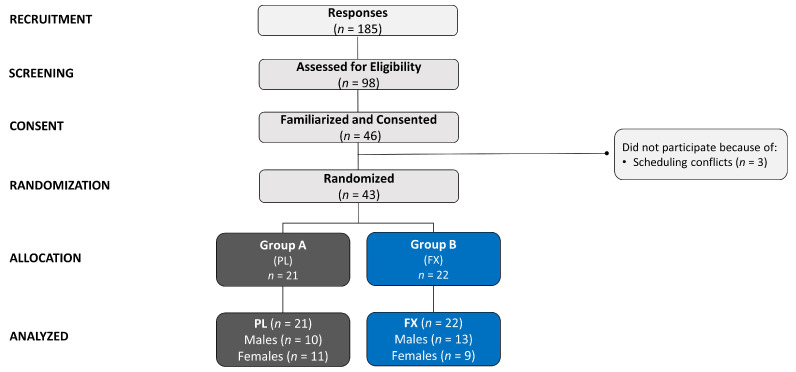
The Consolidated Standards of Reporting Trials (CONSORT) diagram for participant recruitment, screening, consent, randomization, allocation, and analysis of the treatment groups. Unblinding of the treatment groups revealed that Group A was the placebo (PL) and Group B was the fucoxanthin (FX) treatment.

Figure 1 displays a Consolidated Standards of Reporting Trials (CONSORT) schema. Overall, 185 individuals inquired about the study advertisements and were evaluated for eligibility. A total of 98 individuals passed the online pre-screening and were invited to familiarization sessions. Forty-six individuals were familiarized and gave written informed consent. Three participants had scheduling conflicts that prevented them from completing this study. Therefore, 43 individuals were enrolled and randomized into their respective study groups, where 21 participants were allocated to the placebo (PL) group and 22 to the fucoxanthin (FX) group. Data from 43 participants were statistically analyzed. 

### 2.3. Testing Sequence

**Figure 2:**
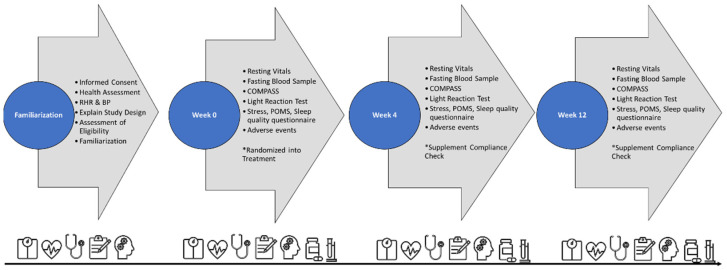
Testing order and timeline. BP = blood pressure, COMPASS = Computerized Mental Performance Assessment System, CPXT = cardiopulmonary exercise test, DEXA = dual-energy X-ray absorptiometry, REE = resting heart rate, RHR = resting heart rate, POMS = Profile of Mood States, 1RM = one repetition maximum.

Figure 2 displays the testing order for the familiarization session and all experimental sessions at 0, 4, and 12 weeks. During the familiarization session, the eligible participants who consented to partake in this study had their baseline testing session scheduled and were instructed to fast (12 h) prior to all testing sessions, record four-day food logs, and refrain from any atypical caffeine or other stimulant consumption (48 h). During baseline testing, the participants returned their four-day food logs and had their resting measures assessed, including weight, height, resting blood pressure (RBP), resting heart rate (RHR), and age-associated cognitive impairment. Then, the participants completed a series of questionnaires, including Cohen’s Perceived Stress Scale, Profile of Mood States (POMS), Leeds Sleep Evaluation, the Bond–Lader Mood Rating Scale, and self-reported side effects. Then, a venous blood sample (≈20 mL) was obtained following standard venipuncture procedures. Lastly, the participants performed cognitive function and light reaction tests. Then, the participants were supplemented with their assigned treatments for 12 weeks. Baseline testing procedures were repeated after week 4 (consistent with a one-month dietary supplement supply) and 12 (to assess a longer intervention). The participants then began supplementation and repeated the experimental testing after 4 and 12 weeks. The participants aimed to maintain their routine diets and physical activity levels during this study and replicate their initial 4-day diets before the testing session.

### 2.4. Participant Familiarization Session

After responding to the advertisements, interested individuals were asked to complete a pre-qualification questionnaire to assess eligibility for a familiarization session. The eligible participants visited the ESNL for the familiarization session, wherein the participants reviewed the study procedures, provided written informed consent, completed health and medical histories, and underwent a physical examination consisting of assessments for height, weight, RHR, and RBP, as well as an age-associated cognitive impairment assessment. The participants practiced the cognitive function tests three times during the familiarization session to familiarize themselves with the tests and establish test-to-test reliability. Following the physical examination, instructions were provided on recording the four-day food logs using the smartphone application MyFitnessPal. The participants practiced all cognitive tests three times to establish reliability. 

### 2.5. Randomization

To keep the supplements randomized and administered in a double-blind manner, the study sponsor used Metlab software version R2021b (Mathworks, Natick, MA, USA) to create a randomization number that was printed on the study product box. The randomized coding was allocated so that age, body fat percentage, BMI, and sex could be used to counterbalance the participants into treatment groups. The participants were instructed to consume either four powder-encased capsules per day of a placebo or a microalgae extract, which contained fucoxanthin (FX), for 12 weeks. 

### 2.6. Supplementation Protocol

The participants were randomly allocated to take supplements containing a placebo or FX for 12 weeks. FX was in powder capsules with 275 mg of microalgae extract from *PT*, containing 0.8% of FX (Brainphyt™, Microphyt, Baillargues, France). The matching PL were color-matched, powered-encased capsules with 275 mg of maltodextrin. The participants ingested four capsules daily for a daily FX dose of 8.8 mg/d or placebo. The study dosages were aligned with United States Food and Drug Administration-approved guidelines. The placebo was manufactured to mimic the appearance and taste of the experimental supplement. The product manufacturers issued a certificate of analysis verifying the absence of contaminants and the dosage. The participants started their supplementation on the first training day following the baseline testing and consumed their treatment daily at lunchtime with water (8 oz). Blister packets were used for supplement distribution, and the participants were instructed to store their supplements at 4 °C. Supplementation compliance was assessed at each testing session, as well as periodic check-ins via emails.

## 3. Procedures

### 3.1. Cognitive Screening

Based on the National Institute of Mental Health criteria, two cognitive tests were performed to confirm age-associated memory impairment (AAMI). The Mini-Mental State Examination (MMSE) was conducted to verify the absence of dementia. A score between 25 and 30 indicates a questionably significant degree of impairment; 20 and 25 mild impairment, 10 and 20 moderate impairment, and 0 and 10 severe impairment. MMSE possesses good test–retest reliability (0.80–0.95). The Memory Complaint Questionnaire (MAC-Q) was conducted to confirm age-related cognitive function inconvenience [47]. A score of 25 or higher indicates subjective memory complaints.

### 3.2. Diet Assessment

To maintain dietary consistency, each participant kept a record of their food and calorie-containing beverage intake for four days before each experimental session using the smartphone application MyFitnessPal Calorie Counter version 21.8.0 (MyFitnessPal, Inc., Baltimore, MD, USA) [48] or by keeping the provided paper meal logs. A total of four days (i.e., three weekdays and one weekend day) of diet records were evaluated using the ESHA Nutrition Analysis Software, Food Processor (Version 11.14.9, Nutrition Research, Salem, OR, USA) [49].

### 3.3. Anthropometrics and Hemodynamics 

A Health-O-Meter Professional 500KL self-calibrating digital scale (Pelstar LLC, Alsip, IL, USA; ±0.02 kg) was used to measure height (cm) and weight (kg). After 6 min of rest, RHR and RBP, both systolic (SBP) and diastolic (DBP), were measured following standard procedures and a digital blood pressure device (Connex^®^ ProBP™ 3400; Welch Allyn, Tilburg, The Netherlands).

### 3.4. Cognitive Function Assessment

Cognitive function was assessed via Computerized Mental Performance Assessment System (COMPASS) software (Version 6.0, Northumbria University, Newcastle upon Tyne, UK). The COMPASS cognitive function assessments included the Corsi Block Task Test, digit vigilance, choice reaction time, word recall, picture recognition, word recognition, and the Stroop color–word test, and previously described tests and methods were used [50]. Briefly, the word recall task test measured episodic memory by recalling and documenting words within a specified timeframe [51]. The word recognition task and picture recognition test were used to evaluate episodic memory by distinguishing target stimuli from decoys [52]. The choice reaction time task test measured response speed and vigilance through prompted arrow-direction identifications [53]. The digit vigilance task evaluated attention and vigilance by prompting responses to varying numerical sequences displayed on-screen [54]. The Corsi Block Task Test evaluated attention and vigilance and required the participants to memorize and accurately reproduce sequences of blue squares presented on a grid [55]. Finally, the Stroop color–word task test was used to assess cognitive attention and processing ability by presenting color-naming challenges, with the participants required to identify the font color of color-named words [56]. Each of these tests has been validated and extensively used to assess various aspects of cognitive function (e.g., working, secondary and spatial memory, reaction time, vigilance, attention, executive function, and mental fatigue) [57,58].

### 3.5. Light Reaction Test Assessment

NeuroTracker Pro using NeurotrackerX (NTX) version 2020 software (Montreal, QC, Canada) was used to assess the reaction performance of light-tracking. This test evaluated perceptual–cognitive skills, as reported in previous studies using similar study procedures [45,59]. Briefly, each participant completed three CORE assessments (3 sessions), each consisting of 20 trials lasting 8 s each. A 3D DLP projector (Optoma Corp., New Taipei City, Taiwan) was used to display the NeuroTracker Pro system. A Zephyrus GX501 gaming laptop (AsusTek Computer Inc., Taipei, Taiwan) was utilized to operate the system with a wireless Logitech G PRO gaming mouse (Logitech Europe S.A., Lausanne, CHE, Switzerland). Throughout the test, the participants were equipped with BOBLOV JX-30 3D DLP-link active shutter glasses (Shenzhen Technology Co., Ltd. in Shenzhen, Guangdong, China). The test–retest coefficient of variation (Cv) for correctly recognizing targets from this test was 6.5%.

### 3.6. Stress, Sleep, and Mood Assessment

Cohen’s Perceived Stress Scale (PSS) was used to evaluate the participants’ perceptions of stress. Specifically, the scale gauged the participants’ perceptions of unpredictability, lack of control, and excessive demands in their lives. Questions within the PSS inquired about the participants’ thoughts and emotions experienced over the preceding month, wherein the participants were asked to indicate the frequency of experiencing specific emotional states in each instance. The test–retest reliability of the PSS was reported to be >0.70 [60]. The Leeds Sleep Evaluation Questionnaire (LSEQ) was used to evaluate the impact of the assigned treatment on sleep quality [61]. The participants responded to ten questions categorized across four distinct subscales as follows: “ease of getting to sleep”, “perceived quality of sleep”, “ease of awakening from sleep”, and “integrity of behavior following wakefulness”. Each question required the participants to indicate their responses by placing a vertical mark on a designated answer line, which spanned 80 mm in length. Cronbach’s alpha coefficients range between 0.78 and 0.92 [62]. Additionally, the Profile of Mood States (POMS) 65-item questionnaire was used to assess mood state changes. The ratings on the POMS questionnaire were grouped into six domains (i.e., confusion, anger, fatigue, depression, tension, and vigor). Vigor scores were subtracted from the sum of scores for confusion, anger, fatigue, depression, and tension to calculate the total mood disturbance score (TMDS). The POMS is a valid and routinely used assessment of mood states [63,64].

### 3.7. Blood Collection and Analysis

Blood samples were collected following standard procedures into two 7.5 mL BD Vacutainer^®^ serum separation tubes (SSTs) and one 3.5 mL BD Vacutainer ethylenediaminetetraacetic acid (EDTA) tube (Becton, Dickinson and Company, Franklin Lakes, NJ, USA). The blood samples were incubated for 15 min at room temperature. Then, the SSTs were centrifuged at 3000 rpm for 10 min using a refrigerated (4 °C) benchtop Thermo Scientific Heraeus MegaFuge 40R Centrifuge (Thermo Electron North America LLC, West Palm Beach, FL, USA). While one SST was aliquoted for serum sample storage, the remaining SST and EDTA samples were transported to the Clinical Pathology Laboratory (Bryan, TX, USA) to be analyzed for comprehensive blood count with differentiation and chemistry panels. The obtained serum was preserved at −80 °C in polypropylene microcentrifuge tubes for subsequent analysis. Implementing this standardized approach guaranteed the conservation of blood samples for future biochemical testing and clinical chemistry analysis. From the serum samples, changes in cytokines and insulin levels were measured with commercial assay kits employed for these analyses. The insulin enzyme-linked immunosorbent assay (ELISA) kit (Alpco Diagnostics, Salem, NH, USA) was used, and absorbance readings were obtained at 450 nm using the BioTek ELX-808 Ultramicoplate reader (BioTek Instruments Inc, Winooski, VT, FL, USA). The levels of serum cytokines, particularly interleukin (IL)-1β, -2, -4, -5, -6, -8, and -10, granulocyte–macrophage colony-stimulating factor (GM CSF), interferon-γ (IFN-γ), and tumor necrosis factor-α (TNF-α), were measured using a commercially available Cytokine Human Magnetic 10-plex Panel. This analysis was performed on a Luminex 200 Instrument System and a Milliplex Analyzer (ThermoFisher Scientific, Vienna, Austria) with xPONENTTM version 4.3 software, following the instructions provided by the company. The prior tests conducted in our laboratory yielded inter-assay coefficient of variation (CV) values ranging from 2.20% to 17.53% and intra-assay CV values ranging from 3.25% to 9.81%.

### 3.8. Side Effects Questionnaire

A self-reported side effects rating assessment was used to evaluate if the participants experienced any side effects due to the assigned treatments. Participants rated the frequency (F) and severity (S) of their side effects experienced during this study (i.e., nervousness, blurred vision, dizziness, heart palpitations, headache, tachycardia, shortness of breath, and any other adverse side effects) using previously described methods [46,65]. The reliability for CVs ranged from 1.2 to 2.6% on responses to these side effects questions in our laboratory [65].

### 3.9. Statistical Analysis

The data were analyzed using Version 29 SPSS^®^ statistical software (IBM Corp., Armonk, NY, USA). The determination of sample size was informed by our prior research assessing the effects of energy drinks and pre-workout supplements [66,67,68], caffeine [45,50,66,67,68,69,70,71,72,73], paraxanthine [50,74,75], ashwagandha [76,77], arginine [59], and the microalgae containing fucoxanthin used in this study [45,46] on cognitive function measures. We also considered reported effect sizes and used the reported means, standard deviations, and statistically significant mean differences to calculate power, assuming an 80% power with a 5–10% standard deviation to the mean and a 5–10% improvement in cognitive test performance. This analysis generally revealed that a sample size of 12–20 per group was sufficient to detect significant differences among the selected variables from the cognitive tests used in this study. General linear model analysis of variance (ANOVA) using a mixed model was used to analyze the data, where between-subject effects were evaluated as separate groups and within-subject effects over time were evaluated using repeated measures. Sphericity was assessed using Mauchly’s test, while skewness and kurtosis statistics were used to test for normality of distributions. Time effects (T) and group × time (G×T) interaction effects were evaluated using Wilks’ Lambda and Greenhouse–Geisser univariate correction tests to adjust for F-value inflation if the assumption of sphericity was violated. The *p*-level (type I error probability) was set at 0.05 or less, while statistical tendencies were revealed when *p*-values ranged between 0.05 and 0.10 with medium to large effect sizes. Partial Eta squared (η_p_^2^) effect size statistics (i.e., small [0.01], medium [0.06], and large [>0.14] effect sizes) [46]. Fisher’s least significant difference (LSD) tests and 95% upper and lower confidence intervals (CIs) for pre-planned contrasts of interest were used to assess pairwise comparisons of means and post hoc tests. This statistical approach provides a comprehensive assessment of multivariate and univariate tests, effect sizes to assess the magnitude of effect, and pairwise comparisons of contrasts of interest to reduce the likelihood of type II error and help researchers decide whether additional research is warranted [78,79]. The clinical significance of the results observed was evaluated via mean changes from baseline with 95% CIs [78,80,81]. Clinical significance was revealed when mean changes and 95% CIs were completely above or below baseline [49]. Data in the tables are displayed as means with standard deviations (SD), while the data in the figures are displayed as mean changes from baseline with 95% CIs (LL, UL). Pearson’s Chi-square analysis was used to assess changes in categorical responses from the questionnaires. Replacement of missing data (<0.6%) was performed using the series means for numerical data [82]. Responses to categorical survey questions (i.e., ordinal data) were replaced using the most frequent response or value method [83].

## 4. Results

### 4.1. Participant Demographics

The participants were primarily educated faculty, staff, and retired professionals from the university community. As seen in Table S1, the participant demographics were 64.3 ± 3.2 years, 171.3 ± 11 cm, 79.8 ± 16.1 kg, a BMI of 27.0 ± 3.9 kg/m^2^, a resting heart of 63.3 ± 8.3 beats/min, a resting SBP of 123.5 ± 16.8 mmHg, and a resting DBP of 75.9 ± 8.6 mmHg. The participants had an MAC-Q score of 27.8 ± 2.3, indicative of subjective memory complaints [21], and an MMSE score of 28.8 ± 0.9, indicating a non-clinically significant impairment [84]. Wilk’s Lambda multivariate analysis demonstrated a significant sex effect (*p* < 0.001, η_p_^2^ = 0.702, large effect) but no group (*p* = 0.160, η_p_^2^ = 0.317, large effect) or group × sex (*p* = 0.882, η_p_^2^ = 0.121, medium effect) within-subject effects. Univariate analysis did not reveal significant differences between groups in demographic variables. Sex differences were observed in height (*p* < 0.001, η_p_^2^ = 0.596, large effect), weight (*p* < 0.001, η_p_^2^ = 0.421, large effect), resting heart rate (*p* = 0.017, η_p_^2^ = 0.138, medium effect), and MAC-Q scores (*p* = 0.013, η_p_^2^ = 0.150, large effect). No sex-by-group interactions were observed in the participant demographics, except that BMI values tended to interact (*p* = 0.071, η_p_^2^ = 0.081, medium effect). 

### 4.2. Cognitive Function Parameter Assessment

#### 4.2.1. Word Recall

**Figure 3:**
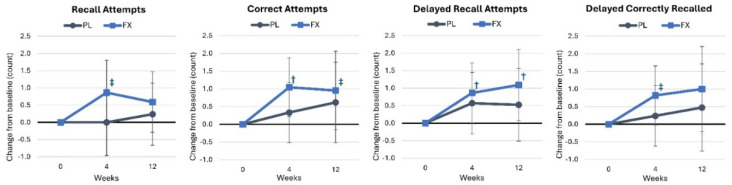
Results of the word recall assessment. Significant changes from baseline are denoted as † = *p* < 0.05, and trends from baseline are denoted as ‡ = *p* > 0.05 to *p* < 0.10.

Table S2 displays the results of the word recall assessment. Wilk’s Lambda multivariate analysis presented a non-significant time effect (*p* = 0.106, η_p_^2^ = 0.079, medium effect) and a non-significant interaction effect (*p* = 0.892, η_p_^2^ = 0.022, small effect). The univariate analysis presented a significant time effect for correct attempts (*p* = 0.050, η_p_^2^ = 0.074, medium effect) and delayed recall attempts (*p* = 0.025, η_p_^2^ = 0.091, medium effect); however, there were no group × time interaction effects. The pairwise comparisons unveiled that baseline and delayed recall attempts were lower in the FX group, and their values increased over time, with no changes seen in the PL group. The analysis of mean changes from baseline is shown in Figure 3. The participants in the FX group experienced improvements in correct attempts (week 4) and delayed recall attempts (4 and 12 weeks), while recall attempts (4 weeks), correct attempts (12 weeks), and delayed correctly recalled values (4 weeks) tended to increase from baseline values in the PL group. No significant changes from baseline scores were found in the PL group. However, no differences were seen between the groups.

#### 4.2.2. Word Recognition

**Figure 4:**
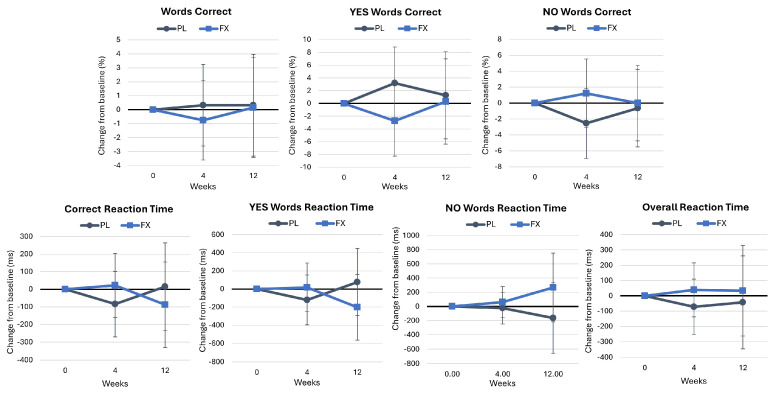
Results of the word recognition assessment. Data are means and 95% confidence intervals. PL = placebo, FX = fucoxanthin.

Table S3 displays the results of the word recognition assessment. Wilk’s Lambda multivariate analysis showed no significant time effects (*p* = 1.000, η_p_^2^ = 0.009, small effect) with no interaction effects observed (*p* = 0.325, η_p_^2^ = 0.080, medium effect). Univariate analysis did not show any significant time or group × time effects. The analysis of percent changes from baseline (Figure 4) revealed similar findings, with no changes observed from baseline or between groups.

#### 4.2.3. Choice Reaction Time

**Figure 5:**
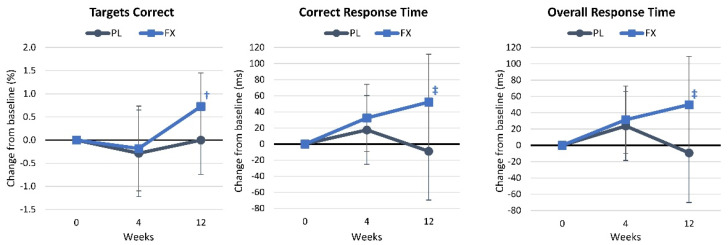
Results of the choice reaction assessment. Data are means and 95% confidence intervals. PL = placebo, FX = fucoxanthin. Significant changes from baseline are denoted as † = *p* < 0.05, and trends from baseline are denoted as ‡ = *p* > 0.05 to *p* < 0.10.

Table S4 presents the results of the choice reaction time assessment. Wilk’s Lambda multivariate analysis showed no significant time (*p* = 0.258, η_p_^2^ = 0.047, small effect) or interaction effects (*p* = 0.374, η_p_^2^ = 0.039, small effect). Similarly, univariate analysis revealed no significant time or group × time effects. Pairwise comparisons revealed that overall and correct response times tended to be higher in the FX group after 4 weeks of supplementation and that the percent targets identified correctly were significantly increased from baseline, while overall and correct response times tended to increase over time with FX supplementation. Choice reaction times did not significantly change over time in the PL group. These changes from the baseline are illustrated in Figure 5.

#### 4.2.4. Picture Recognition Test

**Figure 6:**
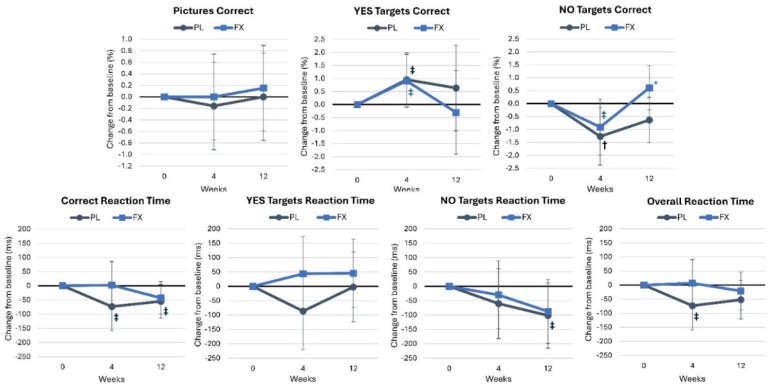
Results of the picture recall assessment. Data are means and 95% confidence intervals. PL = placebo, FX = fucoxanthin. * = *p* < 0.05 difference between groups. Significant changes from baseline are denoted as † = *p* < 0.05, and trends from baseline are denoted as ‡ = *p* > 0.05 to *p* < 0.10.

Table S5 displays the results of the picture recognition assessment. Wilk’s Lambda multivariate analysis demonstrated a significant time effect (*p* = 0.026, η_p_^2^ = 0.136, medium effect) while no significant group × time interaction effects were observed (*p* = 0.569, η_p_^2^ = 0.064, medium effect). Univariate analysis found a time effect in the number of NO targets correctly identified (*p* = 0.042, η_p_^2^ = 0.089, medium effect), while the NO targets reaction time tended to decrease over time (*p* = 0.058, η_p_^2^ = 0.069, small effect). No significant univariate group × time effects were observed. The analysis of mean changes from baseline (Figure 6) revealed that the percentage of NO targets correctly identified was significantly higher after FX supplementation for 12 weeks (1.24 [0.02, 2.4], *p* = 0.047).

#### 4.2.5. Digit Vigilance

**Figure 7:**
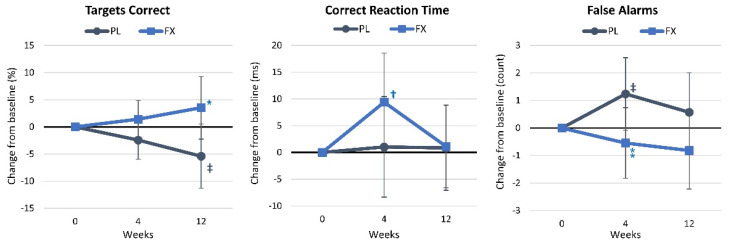
Results of the digit vigilance assessment. Data are means and 95% confidence intervals. PL = placebo, FX = fucoxanthin. * = *p* < 0.05 difference between treatment groups, ⁑ = *p* > 0.05 to *p* < 0.10 difference between treatment groups. Significant changes from baseline are denoted as † = *p* < 0.05, and trends from baseline are denoted as ‡ = *p* > 0.05 to *p* < 0.10.

Table S6 presents digit vigilance test results. Wilk’s Lambda multivariate analysis showed no significant time effects (*p* = 0.583, η_p_^2^ = 0.029, small effect) but a tendency for group × time effects to interact (*p* = 0.052, η_p_^2^ = 0.074, medium effect). Univariate analysis found no significant time effects, while the percentage of targets correctly identified tended to interact (*p* = 0.071, η_p_^2^ = 0.068, medium effect). Figure 7 shows that the percentage of targets correctly identified was significantly higher after FX supplementation for 12 weeks (8.9% [0.7, 17.2], *p* = 0.035), while the number of false alarms tended to be lower after 4 weeks of supplementation in the FX group (−1.8 [−3.6, 0.06], *p* = 0.057). Additionally, the percentage of targets correctly identified at week 12 (−5.4 [−11.3, 0.5], *p* = 0.072) and the number of false alarms at 4 weeks (−1.2 [−2.5, 0.9], *p* = 0.064) tended to decline in the PL group, while correct response reaction time increased after 4 weeks of supplementation in the FX group (9.4 [0.2, 18.6], *p* = 0.045).

#### 4.2.6. Corsi Block

**Figure 8:**
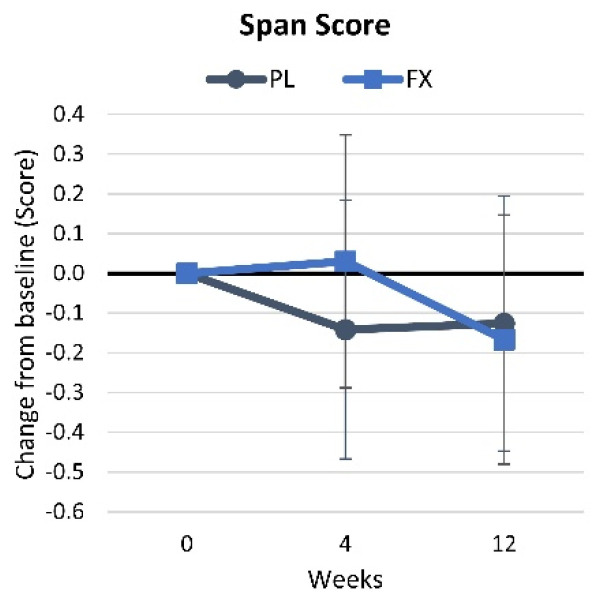
Results of the Corsi block assessment. Data are means and 95% confidence intervals. PL = placebo, FX = fucoxanthin.

Table S7 shows the results of the Corsi Block assessment. No time (*p* = 0.426, η_p_^2^ = 0.021, small effect) or group × time (*p* = 0.607, η_p_^2^ = 0.012, small effect) effects were found for the Corsi Block span score results. Additionally, the mean change from baseline analysis revealed no time or interaction effects for the Corsi Block span scores (Figure 8).

#### 4.2.7. Stroop Test

**Figure 9:**
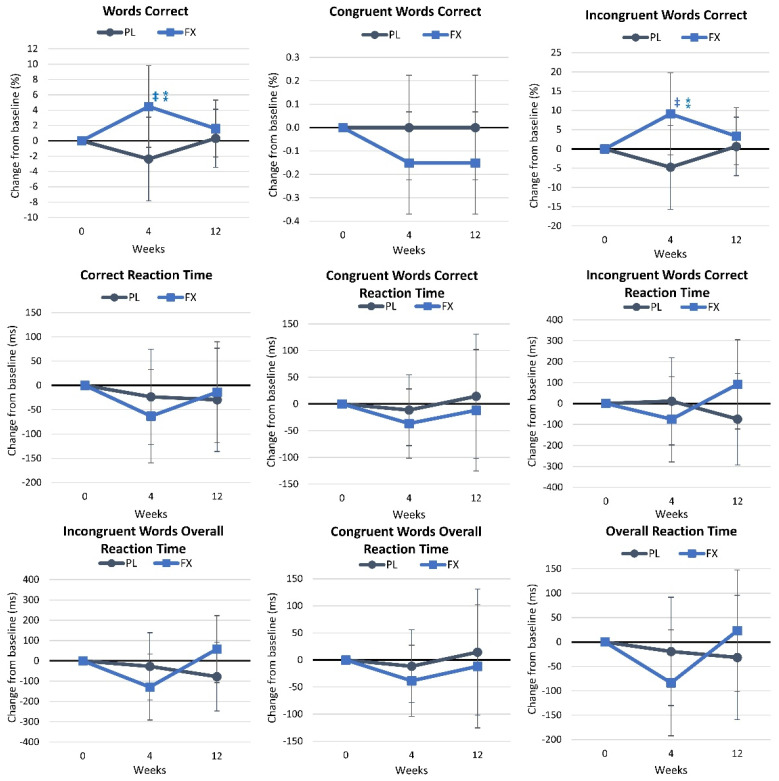
Results of the Stroop color–word assessment. Data are means and 95% confidence intervals. PL = placebo, FX = fucoxanthin. ⁑ = *p* > 0.05 to *p* < 0.10 difference between treatment groups. ‡ = *p* > 0.05 to *p* < 0.10 difference from baseline.

Table S8 shows the results of the Stroop color–word assessment. Wilk’s Lambda multivariate analysis revealed no significant time (*p* = 0.498, η_p_^2^ = 0.093, medium effect) or interaction effects (*p* = 0.610, η_p_^2^ = 0.085, medium effect). Univariate analysis showed no significant time effects. However, the percentage of words correctly identified (*p* = 0.089, η_p_^2^ = 0.060, medium effect) and the percentage of incongruent words correctly identified (*p* = 0.087, η_p_^2^ = 0.060, medium effect) tended to interact. The pairwise comparison revealed that the percentage of words correct (4.5% [−0.8, 9.8], *p* = 0.098) and the percentage of incongruent words correctly identified tended to increase over time (9.1% [−1.6, 19.7], *p* = 0.092) after FX supplementation for 4 weeks, while no time effects were observed in the PL group. These changes are illustrated in Figure 9. Additionally, this figure shows that the percentage of (6.9% [−0.8, 14.5], *p* = 0.077) and incongruent words correct (13.9% [−1.4, 29.1], *p* = 0.074) at 4 weeks of supplementation tended to be higher in the FX group.

### 4.3. Light Reaction Test Results

**Figure 10:**
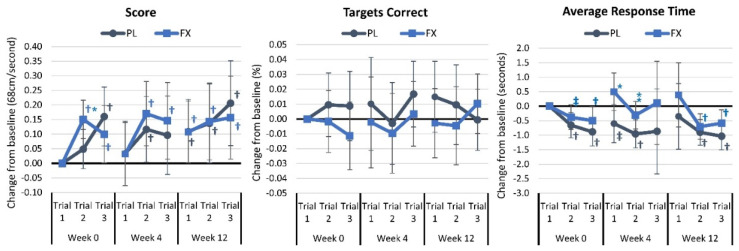
Results of the light reaction assessment. Data are means and 95% confidence intervals. PL = placebo, FX = fucoxanthin. * = *p* < 0.05 difference between groups. ⁑ = *p* > 0.05 to *p* < 0.10 difference between treatment groups. † = *p* < 0.05 differences from baseline. ‡ = *p* > 0.05 to *p* < 0.10 difference from baseline.

Table S9 displays the result of the light reaction assessment. The overall analysis found a time effect (*p* = 0.006, η_p_^2^ = 0.044. small effect) but no group × time interaction (*p* = 0.937, η_p_^2^ = 0.014, small effect). Univariate analysis unveiled a significant time effect in the overall score (*p* = 0.004, η_p_^2^ = 0.077, medium effect) with no other time or group × time interaction effects. Meanwhile, the pairwise comparison analysis revealed some time effects in each group. Targets identified as correct tended to be higher in the PL group at week 12 in trial 1 (0.019% [−0.004, 0.042], *p* = 0.098). Figure 10 presents mean changes from baseline in light reaction results. Light reaction scores increased in both groups over time, with baseline trial 2 scores (0.1 [0.008, 0.196], *p* = 0.034) higher in the FX group. Average response times at 4 weeks in trial 1 (1.1 ms [0.17, 2.02], *p* = 0.021) and trial 3 average response time (0.64 ms [−0.04, 1.33], *p* = 0.063) were higher in the FX group. No significant differences were observed over time or between groups in the number of targets identified as correct.

### 4.4. Psychological Assessment

Table S10 shows the results of the Perceived Stress Scale. Chi-squared analysis revealed that the participants in the FX group had reduced perceptions about how often they were angered by things outside of their control after 12 weeks (*p* = 0.013) and tended to improve perceptions about how often they felt nervous and “stressed” (*p* = 0.084) after 4 weeks, and how often they felt they could not cope with all of the things they had to complete (*p* = 0.085).

Table S11 presents sleep quality responses. Multivariate analysis revealed no significant time (*p* = 0.155, η_p_^2^ = 0.157, large effect) or group × time (*p* = 0.109, η_p_^2^ = 0.166. large effect) effects. Univariate analysis revealed a significant time effect in perceptions about the quality of sleep (*p* = 0.052, η_p_^2^ = 0.069, medium effect), with those in the FX reporting a tendency to experience an increase in calmness compared with restlessness over time (6.8 [−1.2, 14.5], *p* = 0.093), how they awoke from sleep (*p* = 0.062, η_p_^2^ = 0.069, medium effect), with those in the FX group reporting a significant increase in the ease and amount of time to awaken after 4 weeks (11.0 [1.4, 20.7], *p* = 0.027), and how alert they feel after wakening (*p* = 0.052, η_p_^2^ = 0.070, medium effect), with those in the FX group reporting easier (7.7 [−0.8, 16.2], *p* = 0.075) and shorter times (8.5 [0.8, 16.2], *p* = 0.032) after 4 weeks of supplementation, with a tendency to differ between groups (6.3 {0.4, 13.1], *p* = 0.065).

**Figure 11:**
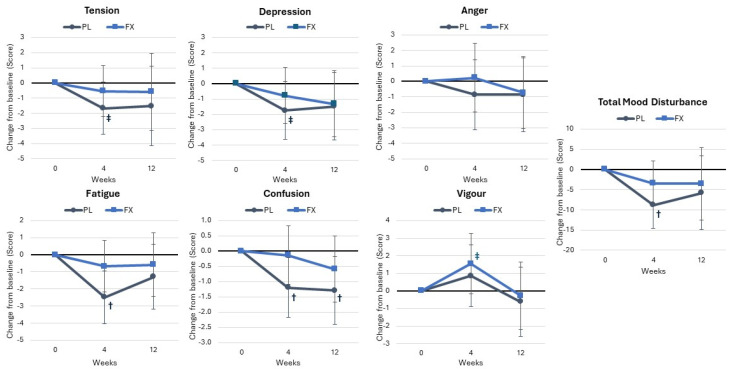
Results of the Profile of Mood States responses. Data are means and 95% confidence intervals. PL = placebo, FX = fucoxanthin. Significant changes from baseline are denoted as † = *p* < 0.05, and trends from baseline are denoted as ‡ = *p* > 0.05 to *p* < 0.10.

The Profile of Mood States responses are shown in Table S12. Multivariate analysis of all POMS variables revealed no significant time (*p* < 0.151, η_p_^2^ = 0.101, medium effect) or interaction effects *p* = 0.943, η_p_^2^ = 0.033, small effect). Univariate analysis revealed tendencies for time effects in depression (*p* = 0.098, η_p_^2^ = 0.057, small effect), fatigue (*p* < 0.052, η_p_^2^ = 0.072, medium effect), confusion (*p* = 0.053, η_p_^2^ = 0.070, medium effect), vigor (*p* = 0.070, η_p_^2^ = 0.064, medium effect), and TMDS (*p* = 0.061, η_p_^2^ = 0.072, medium effect). No significant interaction effects were observed. Additionally, no significant differences were noted between the groups. Mean changes from baseline analysis suggested that those in the PL group had fewer perceptions of tension, depression, fatigue, and confusion with lower vigor at 4 weeks with a lower TMDS from baseline, with no differences found between groups (Figure 11).

### 4.5. Health Biomarkers

Tables S13 and S14 present body weight and resting hemodynamic data, respectively. No significant univariate time (*p* = 0.604, η_p_^2^ = 0.010, small effect) or interaction effects (*p* = 0.637, η_p_^2^ = 0.009, small effect) was observed in body weight. A significant overall time effect (*p* = 0.005, η_p_^2^ = 0.109, medium effect), but no significant group × time effect (*p* = 0.306, η_p_^2^ = 0.043, small effect), was observed in the RHR and RBP responses. Univariate analysis showed a time effect in all hemodynamic variables evaluated with no significant interaction effects. Cell blood count analysis (Table S15) found no time (*p* = 0.569, η_p_^2^ = 0.159, large effect) or group × time effects (*p* = 0.224, η_p_^2^ = 0.199, large effect) in red or white cell variables. Univariate analysis revealed some group and time effects, but differences were small and within normal ranges. 

Table S16 presents blood lipid variables. GLM analysis showed a time effect (*p* = 0.050, η_p_^2^ = 0.139, medium effect) but no interaction effects (*p* = 0.431, η_p_^2^ = 0.086, medium effect). Univariate analysis demonstrated that the LDL-HDL ratio decreased over time (*p* = 0.016, η_p_^2^ = 0.103, medium effect), while HDL cholesterol tended to increase (*p* = 0.089, η_p_^2^ = 0.058, small effect) with values more favorably changed over time in the PL group. However, no significant interaction effects or pairwise differences between groups were observed. No overall time (*p* = 0.362, η_p_^2^ = 0.105, medium effect) or interaction effects (*p* = 0.745, η_p_^2^ = 0.074, medium effect) were observed in markers of liver function (see Table S17). Total protein (*p* = 0.090, η_p_^2^ = 0.058, small effect) and alkaline phosphatase (*p* = 0.079, η_p_^2^ = 0.061, moderate effect) tended to increase over time, with no significant interaction effects observed. Pairwise comparison analysis revealed that aspartate aminotransaminase (−3.0 IU/L [−6.1, 0.08], *p* = 0.056) and alanine aminotransaminase (−4.7 IU/L [−9.6, 0.3], *p* = 0.063) levels at 4 weeks and total protein (−0.13 mg/dL [−0.55, −0.07], *p* = 0.013) and albumin (−0.18 mg/dL [−0.35, −0.003], *p* = 0.046) levels at 12 weeks were lower in the FX group compared with the PL group, while alkaline phosphatase levels decreased over time in the FX group. 

The markers of renal function are shown in Table S18. GLM analysis showed no significant time (*p* = 0.198, η_p_^2^ = 0.136, medium effect) or interaction effects (*p* = 0.846, η_p_^2^ = 0.074, medium effect). Univariate analysis found that blood urea nitrogen (BUN, *p* = 0.034, η_p_^2^ = 0.080, medium effect) and the BUN-to-creatinine ratio (*p* = 0.019, η_p_^2^ = 0.093, medium effect) increase over time with no interaction effects. Pairwise comparison analysis found that the BUN-to-creatinine ratio increased after 4 weeks (1.5 [0.03, 3.0], *p* = 0.046) in the FX group, while the glomerular filtration rate tended to be lower after 12 weeks (−9.6 mL/min/1.73 [−20.9, 1.6], *p* = 0.09), although values were within normal clinical ranges. 

**Figure 12:**
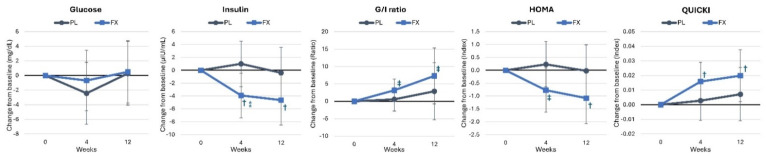
Changes in markers of glucose homeostasis. Data are means and ± 95% confidence intervals. PL = placebo, FX = fucoxanthin, G/I ratio = glucose-to-insulin ratio, HOMA = homeostatic model assessment insulin resistance, QUICKI = quantitative insulin sensitivity check index. ⁑ = *p* > 0.05 to *p* < 0.10 difference between groups. Significant changes from baseline are denoted as † = *p* < 0.05, and trends from baseline are denoted as ‡ = *p* > 0.05 to *p* < 0.10.

Table S19 shows glucose homeostasis-related variables. Overall, GLM analysis showed non-significant time (*p* = 0.181, η_p_^2^ = 0.083, medium effect) and interaction effects (*p* = 0.710, η_p_^2^ = 0.044, small effect). Univariate analysis indicated that quantitative insulin sensitivity check index (QUICKI) values tended to increase over time (*p* = 0.074, η_p_^2^ = 0.064, medium effect), with no other time or interaction effects seen. Pairwise analysis revealed that insulin levels significantly decreased after 4 (−3.91 μIU/mL [−7.4, −0.4], *p* = 0.028) and 12 weeks (−4.63 μIU/mL [−8.58, −0.8], *p* = 0.020) in the FX group, resulting in a higher glucose-to-insulin ratio (4 weeks: 3.14 [−0.13, 6.14], *p* = 0.060; 12 weeks: 7.29 [−0.7, 15.3], *p* = 0.073) and QUICKI score (4 weeks: 0.016 [0.003, 0.029], *p* = 0.019; 12 weeks: 0.02 [0.002, 0.038], *p* = 0.030) and lower homeostatic model assessment insulin resistance (HOMA_IR_) at 4 (−0.77 [−1.63, 0.084], *p* = 0.076) and 12 weeks (−1.081 [−2.071, −0.092], *p* = 0.033), with the difference between groups at 4 weeks tending to be different (−4.92 μIU/mL [−9.9, 0.6], *p* = 0.053), as shown in Figure 12.

**Figure 13:**
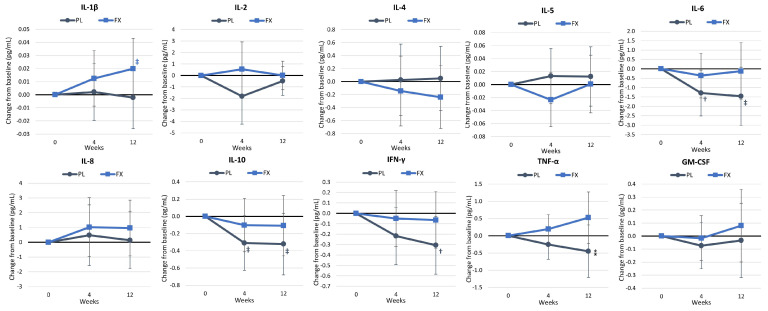
Cytokine and inflammatory marker changes from baseline. Data are presented as means ± 95% confidence intervals. PL = placebo, FX = fucoxanthin, GM-CSF = granulocyte-macrophage colony-stimulating factor, IL = interleukin, IFN-γ = interferon-gamma. Significant changes from baseline are denoted as † = *p* < 0.05, and trends from baseline are denoted as ‡ = *p* > 0.05 to *p* < 0.10. ⁑ = *p* > 0.05 to *p* < 0.10 difference between groups.

Table S20 shows the results of the cytokine and inflammatory markers. Overall, GLM analysis showed no significant time (*p* = 0.580, η_p_^2^ = 0.110, medium effect) or group × time effects (*p* = 0.648, η_p_^2^ = 0.104, medium effect). Univariate analysis showed that IL-10 (*p* = 0.082) and interferon-gamma (INF-γ, *p* = 0.092) tended to change over time, with values tending to decrease in the PL group. No significant interactions were observed in inflammatory or cytokine markers. However, pairwise comparisons found that INF-γ values in the FX group were significantly lower than PL at 4 weeks (−1.33 pg/mL [−0.25, −0.01], *p* = 0.030) and that IL-10, IL-6, and TNF-α increased over time in the PL group. Mean changes from baseline with 95% CIs (Figure 13) revealed that IL-β tended to increase with FX, while IL-6, IL-10, and INF-γ decreased or tended to decrease over time. The pairwise comparison revealed that the mean change in INF-α tended to be higher following the FX treatment (0.97 pg/mL [−0.9, 2.0], *p* = 0.072). compared with the PL group after 12 weeks. 

### 4.6. Safety and Side Effects

Table S21 shows subjective ratings of the frequency and severity of the various monitored side effects. The chi-squared analysis found no differences between groups in the frequency or severity of dizziness, headaches, tachycardia, heart palpitations, dyspnea, nervousness, blurred vision, or other complaints. Reported side effects were infrequent, rated as minimal to slight, and similar between groups. No participant withdrew from this study because of issues with the supplement. These findings and the comprehensive blood panels evaluated indicate that the microalgae extract was well-tolerated and did not cause undesirable side effects in older individuals.

## 5. Discussion

We examined the impact of fucoxanthin supplementation (8.8 mg/d for 12 weeks) from microalgae *PT* extract on several cognitive function parameters, including episodic memory, vigilance, attention, and executive function in adults with perceptions of age-associated memory impairment. In addition, we assessed fucoxanthin’s ability to impact health markers, glucose homeostasis, inflammatory cytokines, resting hemodynamics, subjective profile mood state, sleep, perceived stress questionnaire responses, and self-reported side effects. Our findings indicate that fucoxanthin supplementation can improve (1) some aspects of cognitive function, such as secondary and episodic memory, attention, vigilance, and perceptual–cognitive function; (2) awakeness after sleep, and (3) the insulin response. No difference was found for the adverse events/side effect questionnaire. These findings provide some evidence that fucoxanthin supplementation may help mediate cognitive function and insulin response in response to age-associated cognitive impairment in older adults.

### 5.1. Primary Outcome

We found evidence that fucoxanthin supplementation improved episodic memory (i.e., delayed word recall, word presentation, delayed word recognition, delayed picture recognition, and picture recognition), vigilance and attention (i.e., digit vigilance, simple reaction time, and choice reaction time), and executive function (i.e., the Stroop color–word test) in older adults with self-perceived memory and cognitive decline. As such, the present findings indicate that fucoxanthin supplementation improved (1) working memory via the ability to recall words correctly, (2) episodic memory via maintaining correct and overall reaction time on the picture recognition test, (3) attention and vigilance via identifying correct targets on the choice reaction and digit vigilance tests, and (4) executive function via the ability to identify correct words and incongruent words on the Stroop color–word test. We did not identify differences between the groups for the word recognition test (secondary memory assessment), the Corsi Block test (working and spatial memory assessment), or the light reaction test (assessment of reaction time, reasoning, and vigilance).

Previous reports suggest that fucoxanthin can cross the blood–brain barrier and confer antioxidant and anti-inflammatory effects on the host [37,38], improving cognition and executive function. The present study’s findings first demonstrate that fucoxanthin enhanced the working and episodic memory indices, as shown by the mean change from baseline scores on the word recall and picture recognition tests. The participants supplementing with FX demonstrated improvements in correct attempts (week 4), delayed recall attempts (4 and 12 weeks), recall attempts (4 weeks), correct attempts (12 weeks), and delayed correctly recalled values (4 weeks) compared with the placebo on the word recall assessment. Regarding the picture recognition test, the percentage of NO targets correctly identified was higher for the FX group after 12 weeks than the placebo group. Given that delayed memory (the capacity to store and recall knowledge) is important for everyday tasks [85], especially among older adults, identifying nutritional strategies to combat the deterioration of these cognitive function parameters is critical. Second, the mean change from baseline scores on the choice reaction time and digit vigilance assessments demonstrate that fucoxanthin improved attention and vigilance. The participants’ abilities to correctly identify choice reaction time test targets were higher (week 12) for the FX group. In addition, the FX group had more targets correctly identified (week 12) and fewer false alarms (week 4). Perceptual cognitive function is essential for tasks that include attention, focus, and processing of sensory information in daily activities. These data demonstrate that fucoxanthin enhanced perceptual–cognitive function. Lastly, the mean change from baseline scores for the Stroop color–word test showed that the FX group had higher percentages of correct words and incongruent correct words identified (week 4) than the placebo group. These findings indicate that the intervention may have improved participants’ capacities to handle cognitive interference and suppress irrelevant information, which suggests an enhancement in cognitive flexibility and executive function [86]. Together, these findings provide some evidence that 12 weeks of supplementation with fucoxanthin can improve working and episodic memory parameters, attention and vigilance, and executive function. Previously, we showed that acute and 30-day fucoxanthin supplementation (440 mg of *PT* extract containing 1% or 4.4 mg of FX) in combination with guarana (500 mg containing 40–55 mg of caffeine) improved reasoning, learning, attention shifting (cognitive flexibility), executive control, and impulsiveness among college-aged e-gamers [45]. The present results support fucoxanthin’s ability to improve cognitive function parameters and suggest that it may result in enhanced cognitive vigor and improved quality of life for older persons experiencing age-associated cognitive impairment.

A number of studies have evaluated the effects of diet and various nutritional interventions on cognitive function in individuals with age-associated cognitive decline or neurodegenerative diseases [16,23,28,29,87]. For example, several studies have reported that adherence to a Mediterranean diet (1.0–10.6 years) was associated with slowing cognitive decline [12,29,88,89,90,91,92,93,94,95] and less brain atrophy [96]. Omega-3 fatty acid supplementation has been reported to improve cerebral blood flow in older adults [19], prevent atrophy in Alzheimer’s disease-related brain regions in MCI patients administered 2.2 g/d for six months when combined with aerobic exercise and cognitive stimulation [17], and improve cognition in elderly individuals consuming 2.2 g/d of for 12 weeks [15]. There is also evidence that folic acid [20,21] and vitamin D [22] supplementation may affect cognition in individuals with neurodegenerative diseases. Ma and coworkers [97] reported that folic acid supplementation (400 μg/day for six months) in older adults with MCI improved several cognitive function test scores. Jia et al. [98] reported that dietary supplementation with vitamin D (800 IU/day for 12 months) improved cognitive function and decreased Aβ-related biomarkers in patients with Alzheimer’s disease. Additionally, several studies have reported the effects of dietary ingestion of a yogurt-like drink containing omega-3 fatty acids, choline, phospholipids, folic acid, antioxidants, and vitamins and minerals [23,24,25]. Other nutritional interventions have been studied in an attempt to help maintain cognitive function in individuals experiencing memory issues or diagnosed with MCI and/or dementia [7,16,23,26,27,28,29,30]. For instance, Thaung Zaw and colleagues [99] found that 75 mg of resveratrol improved overall cognitive performance on the National Institutes of Health Toolbox assessment, Rey’s Auditory Verbal Learning Test, Forward Spatial Span Test, and Trail Making Task among middle-aged women. Foroumandi et al. [100] assessed the impacts of 5 cc of fenugreek seed extract in a cohort of patients with moderate to mild Alzheimer’s disease and reported favorable effects on memory and quality of life. 

To our knowledge, the present study is the first to demonstrate that fucoxanthin improves some aspects of cognitive function among an older adult population with perceptions of cognitive decline. As individuals age, impaired cognitive function can present difficulties in everyday life (e.g., managing finances, driving safely, and preparing meals). Attention control and vigilance deficits can serve as early markers of cognitive dysfunction [101], and executive dysfunction (i.e., decreased inhibitory control, working memory, and cognitive flexibility) can result in a reduced sense of time, challenges in transitioning among tasks, and difficulty in managing impulses, planning, and sequencing time [102]. Early identification and intervention, when cognitive deficits begin to be noticed, may be critical to maintaining cognitive function as we age, delaying cognitive decline, and reducing healthcare costs associated with treating these conditions [103,104].

### 5.2. Secondary Outcomes

Our secondary aims targeted fucoxanthin’s ability to impact cardiometabolic health and clinical blood markers, the insulin response, inflammatory cytokines, resting hemodynamics, self-reported side effects, subjective profile mood state, sleep, and perceived stress questionnaire responses. The rationale was based on reports demonstrating that carotenoids have antioxidant and anti-inflammatory properties and the potential to impact mood states, sleep [105], and perceived stress. The participants of the present study who were supplemented with fucoxanthin saw improvements in Cohen’s Perceived Stress Scale and Leeds Sleep Evaluation Questionnaire results. The FX group reported reduced perceptions regarding how often they were angered by things that were outside of their control (week 12), as well as improved perceptions about how often they felt nervous and stressed and how often they felt that they could not cope with all the things that they had to do (week 4). Furthermore, the FX group reported increased calmness to restlessness and greater ease of awakening after sleeping, with better alertness than the placebo group. We noted no difference between the groups in the Profile of Mood States assessment. Lack of sleep is associated with fatigue, cognitive impairment, and mood disturbance [106], and sleep quality is related to systemic inflammation [107]. Previous research has shown that sleep disorders or low sleep quality can negatively affect memory consolidation, attention, and executive function [108,109], and sleep disturbances are linked to impairments in memory consolidation, attention, and executive function [110,111]. Fucoxanthin may affect sleep quality [112] and alleviate some of the negative effects of cognitive impairment in older adults. Our findings provide some evidence that fucoxanthin may favorably impact perceptions of sleep quality. 

There was also evidence that fucoxanthin supplementation improved insulin sensitivity. Mean change from baseline values for insulin and HOMA_IR_ were lower, while the glucose-to-insulin ratio and QUICKI scores were higher for the FX group at weeks 4 and 12. Previous research has demonstrated that fucoxanthin can enhance the expression of GLUT 4, reduce elevated blood sugar levels, and lower adipocytokine levels associated with insulin resistance in white adipose tissue [113,114]. López-Ramos et al. [115] reported that fucoxanthin supplementation (12 mg/d for 12 weeks) in middle-aged patients with metabolic syndrome increased insulin secretion and tended to improve insulin sensitivity. Conversely, we previously reported that fucoxanthin supplementation (4.4 mg/d for 12 weeks) did not improve insulin sensitivity among younger, overweight females [46]. Differences in age and/or dosage may explain this discrepancy in results. Importantly, insulin dysregulation is associated with cognitive disease and disorders, such as Alzheimer’s disease, wherein insulin resistance can exacerbate the accumulation of amyloid-beta plaques and tau tangles [116]. Moreover, insulin plays a vital role in preserving neuronal viability, synaptic plasticity, and memory formation, underscoring its significance in maintaining cognitive well-being [117]. Targeted therapies that address insulin resistance offer potential strategies for promoting healthy brain aging and reducing the risk of neurodegenerative disorders in older adults. Our findings underscore this potential therapeutic property of fucoxanthin, considering the improvements in cognition and insulin sensitivity, which may preserve cognitive function in aging adults with mild cognitive impairment (MCI).

We also assessed the impact of fucoxanthin on resting inflammatory cytokine concentrations. Interestingly, we found some evidence of inflammation in the FX group, as noted by the increased mean change from baseline values for IL-β (week 12), whereas the placebo group experienced decreases in IL-6, TNF-α, and IL-10 over time. Cytokines are related to cognitive function, and fucoxanthin has been suggested to alleviate cognition-related inflammation [118,119]. It is important to note that (1) our subjects had relatively low levels of inflammation and cytokines within a normal range at the beginning of this study, indicating good overall health [120], and (2) we did not assess inflammatory responses to a stressor. Fucoxanthin has been purported to impact inflammatory responses favorably. For example, Maury et al. [40] reported that *PT* feeding reduced brain inflammation and oxidative stress while improving cognitive function in an aging mouse model injected with chronic D-galactose intoxication as a way to impair cognitive function by increasing inflammatory and oxidative stress. Future work is warranted to understand how fucoxanthin supplementation may affect inflammation, oxidative stress, and/or cognitive function when exposed to stressors like intense exercise. Regarding health and safety, we did not note any effects of fucoxanthin on body weight, which mirrors the findings of our recent study. However, it is important to note that the present study was not targeted at weight loss, whereas past work has explored fucoxanthin’s ability to augment weight loss and change body composition. Furthermore, the clinical blood markers, including whole blood cell counts, renal and liver function enzymes, and blood lipids, were unaltered by the supplementation and within normal ranges. Lastly, fucoxanthin supplementation appeared to be well-tolerated, as noted by the lack of self-reported side effects.

### 5.3. Limitations and Future Directions

The strength of this study is that we assessed the effects of fucoxanthin supplementation from microalgae *PT* extract on markers of cognitive function and health in older individuals beginning to experience self-perceived memory and cognitive issues. The limitations included (1) the potential variability among participants in the type and severity of perceptions about memory and cognitive decline; (2) the sample size, which was sufficient in identifying significant differences over time and between groups in several variables but only observed tendencies toward statistical significance with medium to large effect sizes in several other variables that would have likely resulted in significant differences between groups with larger sample size; (3) cognitive test selection; (4) normal variability on performing these types of tests; (5) test fatigue; and (6) the duration of this study (12 weeks). While the tests selected were designed to assess a wide variety of cognitive domains, the participants practiced each test to establish reliability, the test sequence was randomized, and the present study was carried out in a parallel-arm, double-blind, and cross-over manner, these factors may have influenced results. It is also likely that conducting a longer intervention (e.g., 6–12 months) in a larger cohort of older individuals noticing memory issues or in patients with diagnosed cognitive issues (e.g., MCI and/or dementia) may have yielded clearer benefits, particularly since the progression of pre-symptomatic to symptomatic stages can take years. 

We have several suggestions for future research based on our work in this area. First, investigating individuals progressing from pre-symptomatic to symptomatic levels of cognitive impairment and neurodegenerative disease may provide additional insight into the potential benefit of fucoxanthin supplementation on cognitive function. Second, the participants in the present study were healthy with normal levels of inflammation. Additional work should evaluate the effects of fucoxanthin supplementation among individuals with higher levels of inflammation and/or oxidative stress or in response to physical and/or cognitive stress. Additionally, additional research should evaluate whether there may be a dose–response effect on inflammation, oxidative stress, markers of Alzheimer’s disease progression (e.g., amyloid-β (Aβ) pathway (A), tau-mediated pathophysiology (T), and neurodegeneration (N) or AT/N classifications) [121], structural and functional neuroimaging, and cognitive function in younger and older populations with and without perceived or medically treated cognitive impairment, as well as how risk factors to MCI and cognitive neurodegeneration diseases may affect the results. Future research should evaluate whether longer periods of supplementation with and without daily cognitive stimulation activities may promote greater effects. Incorporating an exercise training intervention could also provide additional benefits by promoting increased cerebral blood flow [59,77,78,79,80]. Aerobic exercise has been reported to enlarge the hippocampus, which is crucial for memory formation, by increasing the secretion of neurotrophic substances that promote neurogenesis, vascularization, and synaptic plasticity [81,82]. These processes are significantly influenced by brain-derived neurotrophic factors (BDNFs) and growth factors, such as insulin-like growth factor 1 [83,84]. Lactate, which can cross the blood–brain barrier, also stimulates nuclear protein SIRT-1 and inhibits class I HDACs, activating cerebral BDNF expression [85]. To increase BDNF, an exercise regimen with an intensity of 65% of aerobic capacity, performed two to three times per week for at least 40 min, is recommended [84]. Furthermore, resistance exercise has also been reported to enhance cognition [86] by promoting growth hormone and insulin-like growth factor 1, which enhances brain plasticity. Testosterone also promotes neuronal growth and survival. The production of BDNF and lactate supports the survival of existing neurons and stimulates the growth of new neurons and synapses, which are crucial for learning and memory. Lastly, concurrent training has demonstrated positive effects on cognitive function comparable to those of aerobic exercise and more so than resistance exercise alone. Given the findings from our previous study involving fucoxanthin supplementation during an exercise and diet intervention [23], the benefits of concurrent training in preserving muscle mass while reducing fat mass are noteworthy. Investigating the synergistic effects of fucoxanthin and exercise warrants further exploration, particularly for enhancing cognitive function among the elderly. Additional objective measures could also be included in a longer-term study to better assess activity levels, sleep patterns and quality, and cognitive function. Finally, additional research should evaluate whether co-ingestion of fucoxanthin with other nutrients that have shown promise in delaying cognitive decline in MCI and Alzheimer’s patients (e.g., omega-3 fatty acids, folic acid, vitamin D, etc.) may provide synergistic or additive benefits. 

## 6. Conclusions

The present findings provide some evidence that FX supplementation may improve attention, working/secondary memory, vigilance, accuracy, and executive function. There was also evidence that FX promoted more positive effects on insulin sensitivity and perceptions about sleep quality with no adverse effects on clinical blood panels (i.e., whole blood cell counts, renal and liver function enzymes, blood lipids, and inflammatory cytokines) or perceived side effects. These findings suggest that fucoxanthin supplementation may play a role in mitigating early signs of cognitive decline in healthy, older individuals and add to a growing body of evidence that nutritional interventions can affect cognitive function in older individuals. Identifying effective nutritional strategies may be a cost-effective way to delay the cognitive decline associated with aging and/or an adjunctive nutritional strategy for patients being treated for MCI or neurodegenerative diseases. Given the growing aging population, this nutritional strategy may help reduce medical costs. However, more research is needed to substantiate these findings and to determine whether long-term fucoxanthin supplementation may help delay the onset of mild cognitive impairment in individuals who are beginning to experience signs of cognitive impairment and/or individuals with clinically diagnosed MCI and neurodegenerative diseases. 

## Data Availability

Data and statistical analyses are available for non-commercial scientific inquiry and/or educational use upon request to the corresponding author as long as the use of data does not violate IRB restrictions and sponsored research agreements and the authors and sponsors of this work are appropriately acknowledged.

## Supplementary Materials

The following supplemental materials are available online at https://www.mdpi.com/article/10.3390/nu16172999/s1, Table S1: Participant demographics; Table S2: Results of the word recall test; Table S3: Results of the word recognition test; Table S4: Results of the choice reaction test; Table S5: Results of the picture recognition test; Table S6: Results of the digit vigilance test; Table S7: Results of the Corsi block test; Table S8: Results of the Stroop color–word test; Table S9: Results of the light reaction test; Table S10: Results of the perceived stress scale test; Table S11: Results of the Leeds sleep evaluation questionnaire test; Table S12: Results of the Profile of Mood States test; Table S13: Body weight changes; Table S14: Heart rate and blood pressure results; Table S15: Cell blood count results; Table S16: Lipid profile results; Table S17: Liver function marker results; Table S18: Renal function results; Table S19: Glucose homeostasis results; Table S20: Inflammatory and cytokine results; Table S21: Frequency and severity of side effects.
